# Leading causes of cardiovascular hospitalization in 8.45 million US veterans

**DOI:** 10.1371/journal.pone.0193996

**Published:** 2018-03-22

**Authors:** Nirupama Krishnamurthi, Joseph Francis, Stephan D. Fihn, Craig S. Meyer, Mary A. Whooley

**Affiliations:** 1 Veterans Affairs Medical Center, San Francisco, California, United States of America; 2 Department of Medicine, University of California San Francisco, San Francisco, California, United States of America; 3 Office of Reporting, Analytics, Performance Improvement and Deployment, Veterans Health Administration, Washington, D.C., United States of America; 4 Departments of Medicine and Health Services, University of Washington, Seattle, Washington, United States of America; 5 Department of Epidemiology and Biostatistics, University of California San Francisco, San Francisco, California, United States of America; Kurume University School of Medicine, JAPAN

## Abstract

**Background:**

We sought to determine the leading causes of cardiovascular (CV) hospitalization, and to describe and compare national rates of CV hospitalization by age, gender, race, ethnicity, region, and year, among U.S. veterans.

**Methods:**

We evaluated the electronic health records of all veterans aged ≥18 years who had accessed any healthcare services at either a VA healthcare facility or a non-VA healthcare facility that was reimbursed by the VA, between January 1 2010 and December 31 2014. Among these 8,452,912 patients, we identified the 5 leading causes of CV hospitalization and compared rates of hospitalization by age, gender, race, ethnicity, region, year and type of VA healthcare user.

**Results:**

The top 5 causes of CV hospitalization were: coronary atherosclerosis, heart failure, acute myocardial infarction, stroke and atrial fibrillation. Overall, 297,373 (3.5%) veterans were hospitalized for one or more of these cardiovascular conditions. The percentage of veterans hospitalized for one or more of these CV conditions decreased over time, from 1.23% in 2010 to 1.18% in 2013, followed by a slight increase to 1.20% in 2014. There was significant variation in rates of CV hospitalization by gender, race, ethnicity, geographic region, and urban vs. rural zip code. In particular, older, male, Black, non-Hispanic, urban and Continental region veterans experienced the highest rates of CV hospitalizations.

**Conclusions:**

Among 8.5 million patients enrolled in the VA healthcare system from 2010 to 2014, there was substantial variation in rates of CV hospitalization by age, gender, race, geographical distribution, year, and use of non-VA (vs. VA only) healthcare care facilities.

## Introduction

Cardiovascular disease (CVD) is the leading cause of hospitalization in the US and the leading cause of mortality in developed countries[1], accounting for nearly 1 in 3 deaths in the United States[2]. More than ever, effective health care relies on understanding population-level patterns of CVD. Adoption of electronic health records (EHR) and their recent transformation into nationally harmonized big data files make it possible for researchers to characterize population-level trends in health and healthcare. During the past decade, the Veterans Health Administration (VA) has constructed a centrally harmonized Corporate Data Warehouse (CDW) to standardize patient-level data collected from over 140 medical centers and 1200 free-standing outpatient clinics[3]. Because the VA is the largest healthcare system in the United States (US), the CDW provides a unique opportunity to evaluate population-level rates of hospitalization and how they differ across demographic groups.

Therefore, we sought to 1) determine the leading causes of cardiovascular (CV) hospitalization, and 2) describe and compare national rates of CV hospitalization by age, gender, race, ethnicity, region, year, and use of non-VA (vs. VA only) healthcare facilities, among U.S. veterans.

## Methods

### Database

We used the VA national Corporate Data Warehouse (CDW) Inpatient, Outpatient and Fee Basis files to extract data for this study. The study was approved by the University of California, San Francisco and San Francisco VA Medical Center institutional review boards under the QUERI (VA Quality Improvement Research and Training Initiative) protocol. Our database contained patient identifiers and the requirement for informed consent was waived by the IRB.

### Patient population and data collection

We identified all unique patients ≥ 18 years old, who accessed the VA health care system between January 1, 2010 and December 31, 2014. “Accessed” was defined as having at least one encounter (inpatient, outpatient, emergency department) recorded at either a VA facility or a non-VA facility that was paid for by the VA. All patients hospitalized for any cause were identified. We then identified patients who had an ICD9 discharge diagnosis code for diseases of the circulatory system (ICD9 codes 390 through 459) and calculated the number of unique veterans hospitalized for each code.

We also obtained demographic information (age, sex, race, ethnicity, rural/urban status) and information on healthcare visits (date of visit, location of VHA facility, VA/non-VA care) for all patients. We used the VA urban/rural crosswalk to determine urban/rural status based on the patient’s home address zip code[4]. Race and ethnicity were defined based on Office of Management and Budget (OMB) guidelines. Patients were coded into 5 different US regions (per the Veterans Benefits Administration district definitions[5], accessed Jan 24, 2018) on the basis of their primary address zip code. Veterans were categorized as users of only VA care or users of additional care outside the VA, paid for by the VA (VA and non-VA users).

### Definitions

Cardiovascular (CV) hospitalization was defined as hospitalization due to one or more of the 5 most common cardiovascular conditions. Hospitalization rate was defined as the number of unique veterans per 100 veterans that were hospitalized between January 1, 2010 and December 31, 2014. Previous studies have demonstrated the validity of using VA electronic health records to identify patients with cardiovascular disease[6–9]. We started by identifying patients who had any ICD9 discharge diagnosis code of 390 through 459 (diseases of the circulatory system). We found that the 5 most common circulatory disorder ICD9 discharge diagnosis codes were: 414.01 (coronary atherosclerosis of native coronary artery), 428.0 (congestive heart failure, unspecified), 427.31 (atrial fibrillation), 410.71 (subendocardial infarction, initial episode of care) and 434.91 (cerebral artery occlusion, unspecified with cerebral infarction). We then expanded our definitions to include all ICD-9 codes used by the CMS chronic conditions data warehouse[10] for each of these top 5 conditions (see below). We were unable to find a similar definition of coronary atherosclerosis in the CMS chronic conditions warehouse and therefore included ICD9 codes 414.0x to capture coronary atherosclerosis in a more inclusive manner.

ICD9 codes used:

- Coronary atherosclerosis: 414.0x
- Heart failure: 398.91, 402.01, 402.11, 402.91, 404.01, 404.03, 404.11, 404.13, 404.91, 404.93, 428.x
- Atrial fibrillation: 427.31
- Myocardial infarction: 410.x
- Stroke: 430, 431, 433.01, 433.11, 433.21, 433.31, 433.81, 433.91, 434.00, 434.01, 434.10, 434.11, 434.90, 434.91, 435.0, 435.1, 435.3, 435.8, 435.9, 436

### Statistical analysis

Age-adjusted rates of CV hospitalization (per 100 veterans) over a 5-yr period were calculated in addition to yearly and average annual age-adjusted CV hospitalization rates for all classes of demographic and geographic variables. We separately calculated the proportion of veterans hospitalized for each of the top 5 CV conditions. Multivariate regression models were used to predict the odds of cardiovascular hospitalization. All statistical analyses were carried out using SAS (version 9.3, SAS Institute, Cary, NC) and STATA (version 14, StataCorp, College, TX) statistical packages.

## Results

A total of 8,452,912 unique veterans accessed the VA health care system between January 1, 2010 and December 31, 2014. This cohort predominantly consisted of White (69%), non-Hispanic (84%), male (93%) patients who had an average age of 60 years [Table 1]. Veterans had a similar distribution of rural and urban origins, but there were more veterans living in the Southeast, Midwest or North Atlantic regions than in the Continental or Pacific regions.

**Table 1:** Characteristics of veterans hospitalized vs. not hospitalized for one of the top 5 cardiovascular conditions between 2010 and 2014 *†.

Patient Characteristics		All		Hospitalized		Not Hospitalized	
		N = 8,452,912		N = 297,373		N = 8,155,539	
Age, years (mean ± SD)		60.01 ± 17.63		68.24 ± 11.18		59.71 ± 17.75	
Sex	Female, number (%)	7,045	2.4	7,045	2.4	572,603	7.0
	Male	290,328	97.6	290,328	97.6	7,584,310	93.0
Race	White	223,778	75.3	223,778	75.3	5,625,270	69.0
	Black or African American	47,910	16.1	47,910	16.1	1,156,068	14.2
	Native Hawaiian or Other Pacific Islander	2,108	0.7	2,108	0.7	59,197	0.7
	American Indian or Alaska Native	1,849	0.6	1,849	0.6	53,348	0.7
	Asian	1,330	0.5	1,330	0.5	74,010	0.9
Ethnicity	Not Hispanic	270,873	91.1	270,873	91.1	6,861,580	84.1
	Hispanic	14,751	5.0	14,751	5.0	437,414	5.4
Region	Southeast	61,067	20.5	61,067	20.5	1,585,215	19.4
	Midwest	62,495	21.0	62,495	21.0	1,724,617	21.1
	North Atlantic	57,662	19.4	57,662	19.4	1,793,078	22.0
	Continental	50,453	17.0	50,453	17.0	1,290,610	15.8
	Pacific	47,962	16.1	47,962	16.1	1,383,913	17.0
Rural/Urban Status	Urban	136,450	45.9	136,450	45.9	3,784,017	46.4
	Rural	143,114	48.1	143,114	48.1	3,989,313	48.9
VA Healthcare User Type	VA Only	79,180	26.6	79,180	26.6	5,568,565	68.3
	VA and Non-VA Users	218,193	73.4	218,193	73.4	2,588,348	31.7
Diagnosis	Coronary atherosclerosis	93,380	1.1	93,380	31.4		N/A
	Heart failure	88,769	1.1	88,769	29.9		
	Acute myocardial infarction	61,501	0.7	61,501	20.7		
	Stroke	58,386	0.7	58,386	19.6		
	Atrial fibrillation	45,115	0.5	45,115	15.2		

* All p values (hospitalized vs. not hospitalized) <0.0001 except rural/urban status (p = 0.3967).

^†^ Unknown race: 20,398 (6.9%) hospitalized; 1,188,907 (14.6%) not hospitalized

Unknown ethnicity: 11,749 (4.0%) hospitalized; 857,851 (10.5%) not hospitalized

Unknown region: 17,734 (6.0%) hospitalized, 379,375 (4.7%) not hospitalized

Unknown rural/urban status: 17,809 (6.0%) hospitalized; 383,478 (4.7%) not hospitalized

### Causes of hospitalization

The 5 leading causes of CV hospitalization were coronary atherosclerosis, heart failure, acute myocardial infarction, stroke and atrial fibrillation. Of the 8,452,912 unique veterans, 297,373 (3.5%) were hospitalized for one or more of these 5 CV conditions between 2010 and 2014. Veterans with one or more CV hospitalizations were on average 8.5 years older than those without CV hospitalizations [Table 1].

### Sex

**Fig 1:**
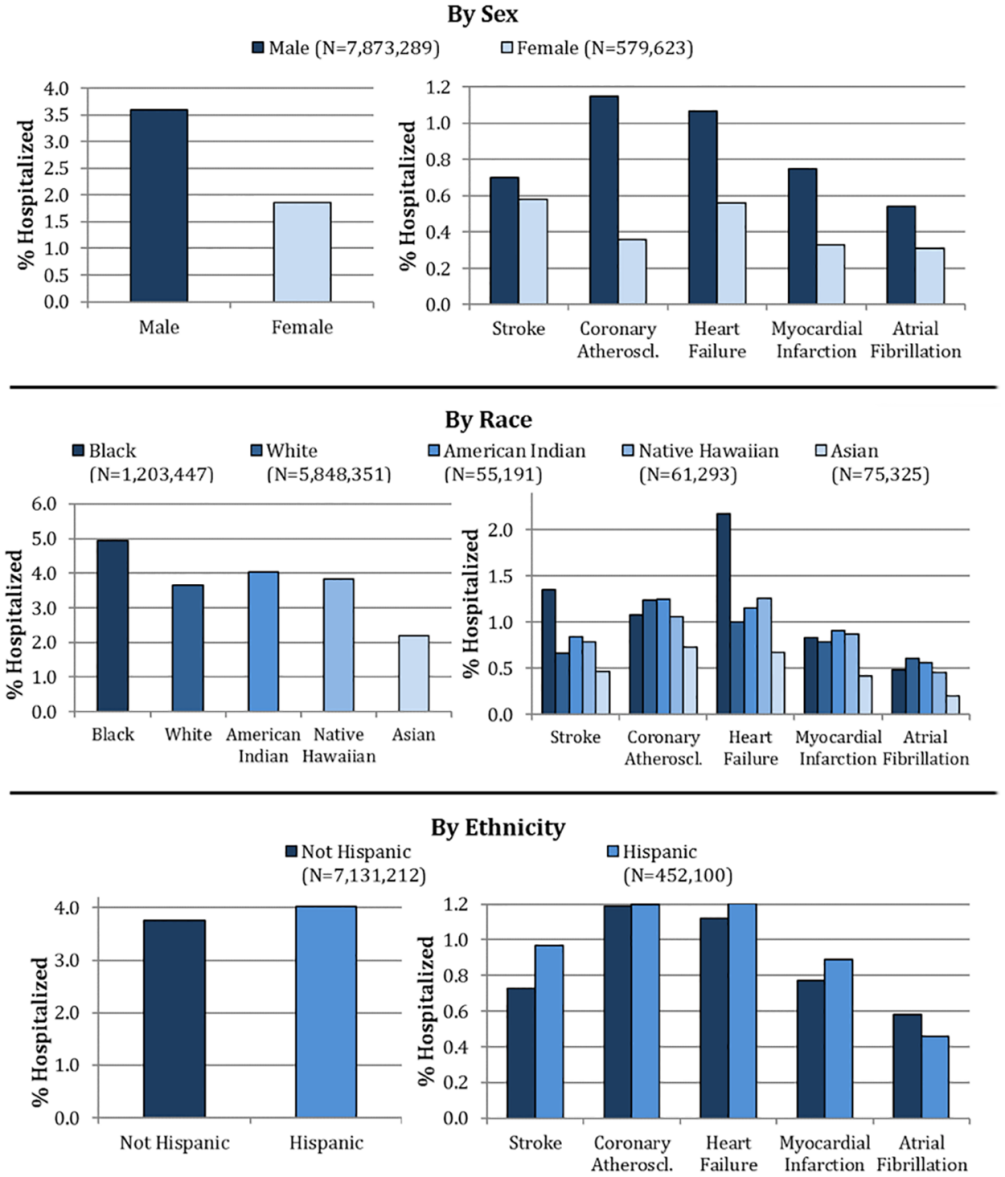
Variation in hospitalization rates by sex, race and ethnicity. Coronary atheroscl.: Coronary atherosclerosis.

On comparison by sex, we found that men were more likely than women to be hospitalized for CVD. During the 5-year period, 3.6% of male veterans experienced one or more CV hospitalizations as opposed to 1.9% of female veterans, adjusted for age [Fig 1]. Annual age-adjusted rates of CV hospitalization were 1.23 per 100 male veterans vs. 0.56 per 100 female veterans [Table 2]. When we analyzed our study sample further by breaking down CV hospitalizations by condition, men were more than twice as likely as women to be hospitalized for each separate CV condition, with the exception of stroke, for which men and women had similar rates [Fig 1]. Multivariate regression models adjusted for other demographics as covariates showed that men had significantly greater odds of CV hospitalization (overall, and by each of the 5 conditions) than women [Tables 3 and 4].

**Table 2:** Age-adjusted annual rates of hospitalization (per 100 veterans) for one or more of the top 5 cardiovascular conditions.

		2010	2011	2012	2013	2014	Avg Annual Rate
Sex	Female	0.57	0.54	0.57	0.57	0.56	0.56
	Male	1.26	1.24	1.22	1.21	1.23	1.23
Race	White	1.25	1.23	1.20	1.18	1.20	1.21
	Black or African American	1.61	1.61	1.58	1.57	1.62	1.60
	Native Hawaiian or Other Pacific Islander	1.15	1.18	1.23	1.23	1.26	1.21
	American Indian or Alaska Native	1.32	1.30	1.34	1.25	1.33	1.31
	Asian	0.73	0.75	0.78	0.81	0.76	0.77
Ethnicity	Not Hispanic	1.27	1.25	1.23	1.22	1.23	1.24
	Hispanic	1.42	1.39	1.28	1.24	1.27	1.32
Region	Southeast	1.33	1.31	1.26	1.23	1.23	1.27
	Midwest	1.16	1.13	1.11	1.10	1.12	1.12
	North Atlantic	1.03	1.02	1.02	1.02	1.07	1.03
	Continental	1.41	1.36	1.37	1.36	1.36	1.37
	Pacific	1.19	1.20	1.20	1.19	1.18	1.19
Rural/Urban Status	Urban	1.24	1.23	1.22	1.21	1.25	1.23
	Rural	1.18	1.16	1.14	1.11	1.12	1.14
VA Healthcare User Type	VA Only	0.53	0.49	0.46	0.45	0.47	0.48
	VA and Non-VA Users	2.46	2.41	2.38	2.34	2.41	2.40

**Table 3:** Age-adjusted and multivariate models evaluating predictors of cardiovascular hospitalization due to one or more of the top 5 cardiovascular conditions ‡.

Predictor Variables		Age-Adjusted OR		Multivariate Model		
		OR	95% CI	OR	95% CI	p value
Age (per 5-year increase)		-	-	1.17	(1.17, 1.17)	<.0001
Sex	Female	Ref.		Ref.		
	Male	1.98	(1.93, 2.03)	2.82	(2.76, 2.89)	<.0001
Race	White	Ref.		Ref.		
	Black or African American	1.37	(1.36, 1.39)	1.26	(1.25, 1.27)	<.0001
	Native Hawaiian or Other Pacific Islander	1.05	(1.00, 1.09)	0.91	(0.87, 0.95)	<.0001
	American Indian or Alaska Native	1.10	(1.05, 1.16)	0.96	(0.92, 1.01)	0.1111
	Asian	0.59	(0.56, 0.62)	0.60	(0.57, 0.64)	<.0001
Ethnicity	Not Hispanic	Ref.		Ref.		
	Hispanic	1.07	(1.06, 1.09)	0.96	(0.94, 0.97)	<.0001
Region	Pacific	Ref.		Ref.		
	Southeast	1.09	(1.07, 1.10)	1.04	(1.03, 1.05)	<.0001
	Midwest	0.96	(0.95, 0.98)	0.99	(0.98, 1.00)	0.1121
	North Atlantic	0.86	(0.84, 0.87)	1.06	(1.05, 1.07)	<.0001
	Continental	1.16	(1.14, 1.17)	1.13	(1.12, 1.14)	<.0001
Rural/Urban Status	Rural	Ref.		Ref.		
	Urban	1.03	(1.02, 1.04)	1.19	(1.18, 1.20)	<.0001
Year	2010	Ref.		Ref.		
	2011	0.98	(0.97, 0.99)	0.96	(0.95, 0.98)	<.0001
	2012	0.97	(0.96, 0.98)	0.94	(0.93, 0.95)	<.0001
	2013	0.96	(0.95, 0.97)	0.93	(0.92, 0.94)	<.0001
	2014	0.97	(0.96, 0.98)	0.96	(0.94, 0.98)	<.0001
VA Healthcare User Type	VA Only	Ref.		Ref.		
	VA and Non-VA Users	6.83	(6.78, 6.89)	4.98	(4.94, 5.02)	<.0001

^‡^ The number of veterans included in the multivariate model was 6,776,493 due to exclusion of unknown race, ethnicity, region and rural/urban status data points

**Table 4:** Five multivariable model evaluating predictors of hospitalization due to each of the top 5 cardiovascular conditions §.

Predictor Variables in Multivariate Model		Coronary atherosclerosis		Heart failure		Acute myocardial infarction		Stroke		Atrial fibrillation	
		OR	95% CI	OR	95% CI	OR	95% CI	OR	95% CI	OR	95% CI
Age (per 5-year increase)		1.09	(1.08, 1.09)	1.29	(1.29, 1.30)	1.12	(1.12, 1.12)	1.17	(1.17, 1.18)	1.16	(1.16, 1.17)
Sex	Female	Ref.		Ref.		Ref.		Ref.		Ref.	
	Male	4.35	(4.13, 4.59)	2.76	(2.63, 2.89)	3.26	(3.08, 3.46)	1.76	(1.68, 1.85)	2.37	(2.23, 2.52)
Race	White	Ref.		Ref.		Ref.		Ref.		Ref.	
	Black or African American	0.80	(0.78, 0.81)	2.07	(2.04, 2.11)	0.96	(0.94, 0.99)	1.84	(1.80, 1.88)	0.66	(0.64, 0.68)
	Native Hawaiian or Other Pacific Islander	0.78	(0.71, 0.84)	1.08	(1.00, 1.16)	0.93	(0.84, 1.02)	1.01	(0.91, 1.12)	0.69	(0.61, 0.78)
	American Indian or Alaska Native	0.91	(0.84, 0.98)	0.95	(0.87, 1.04)	1.00	(0.91, 1.10)	1.10	(0.99, 1.22)	0.89	(0.79, 1.00)
	Asian	0.61	(0.56, 0.68)	0.64	(0.58, 0.70)	0.58	(0.51, 0.66)	0.72	(0.64, 0.82)	0.40	(0.34, 0.47)
Ethnicity	Not Hispanic	Ref.		Ref.		Ref.		Ref.		Ref.	
	Hispanic	0.88	(0.85, 0.91)	1.00	(0.97, 1.03)	1.03	(0.99, 1.07)	1.26	(1.22, 1.31)	0.64	(0.61, 0.67)
Region	Pacific	Ref.		Ref.		Ref.		Ref.		Ref.	
	Southeast	1.23	(1.2, 1.26)	0.92	(0.90, 0.94)	1.00	(0.97, 1.02)	0.98	(0.96, 1.01)	1.11	(1.07, 1.14)
	Midwest	1.05	(1.03, 1.07)	0.93	(0.92, 0.95)	1.01	(0.98, 1.04)	0.93	(0.90, 0.95)	1.04	(1.01, 1.07)
	North Atlantic	1.09	(1.06, 1.11)	1.01	(1.00, 1.04)	1.09	(1.06, 1.12)	0.98	(0.96, 1.01)	1.17	(1.13, 1.20)
	Continental	1.32	(1.29, 1.35)	1.03	(1.01, 1.05)	1.08	(1.05, 1.11)	1.11	(1.08, 1.14)	1.10	(1.06, 1.13)
Rural/Urban Status	Rural	Ref.		Ref.		Ref.		Ref.		Ref.	
	Urban	1.03	(1.02, 1.05)	1.30	(1.29, 1.32)	1.14	(1.12, 1.16)	1.25	(1.23, 1.27)	1.27	(1.24, 1.29)
Year	2010	Ref.		Ref.		Ref.		Ref.		Ref.	
	2011	0.90	(0.89, 0.92)	0.98	(0.96, 1.00)	1.00	(0.97, 1.03)	0.98	(0.96, 1.01)	1.01	(0.98, 1.04)
	2012	0.84	(0.82, 0.85)	0.95	(0.93, 0.97)	1.01	(0.98, 1.03)	0.99	(0.96, 1.02)	1.02	(0.99, 1.05)
	2013	0.77	(0.75, 0.79)	0.98	(0.96, 1.00)	1.00	(0.98, 1.03)	0.98	(0.95, 1.00)	1.02	(0.99, 1.05)
	2014	0.76	(0.75, 0.78)	1.04	(1.02, 1.06)	1.04	(1.01, 1.07)	1.01	(0.98, 1.04)	1.06	(1.03, 1.09)
VA Healthcare User Type	VA Only	Ref.		Ref.		Ref.		Ref.		Ref.	
	VA and Non-VA Users	4.75	(4.68, 4.82)	4.78	(4.71, 4.85)	7.19	(7.04, 7.35)	4.81	(4.71, 4.90)	4.52	(4.43, 4.62)

^§^ The number of veterans included in the multivariate model was 6,776,493 due to exclusion of unknown race, ethnicity, region and rural/urban status data points.

### Race and ethnicity

Blacks had the highest age-adjusted rate of CV hospitalization among all classes of race (4.9%), followed by American Indians (4.02%), Native Hawaiians (3.8%) and Whites (3.7%) [Fig 1]. Among all racial groups, Asians had the lowest age-adjusted rates for all 5 CV conditions. For specific conditions, Blacks had the highest age-adjusted rates for stroke (1.4%) and heart failure (2.2%), while Whites and American Indians had the highest rates for atrial fibrillation and coronary atherosclerosis. This trend was also consistent when adjusted for other demographics, with Blacks demonstrating significantly higher odds of CV hospitalization overall, and for stroke and heart failure, compared to Whites [Tables 3 and 4]. In age-adjusted analyses, rates of CV hospitalization were lower in non-Hispanics vs. Hispanics (3.7% vs 4.0%). However, Hispanics showed significantly lower odds of CV hospitalization compared to non-Hispanics after multivariate adjustment (OR 0.96, 95% CI 0.94, 0.97) [Table 3].

### Urban vs. rural

**Fig 2:**
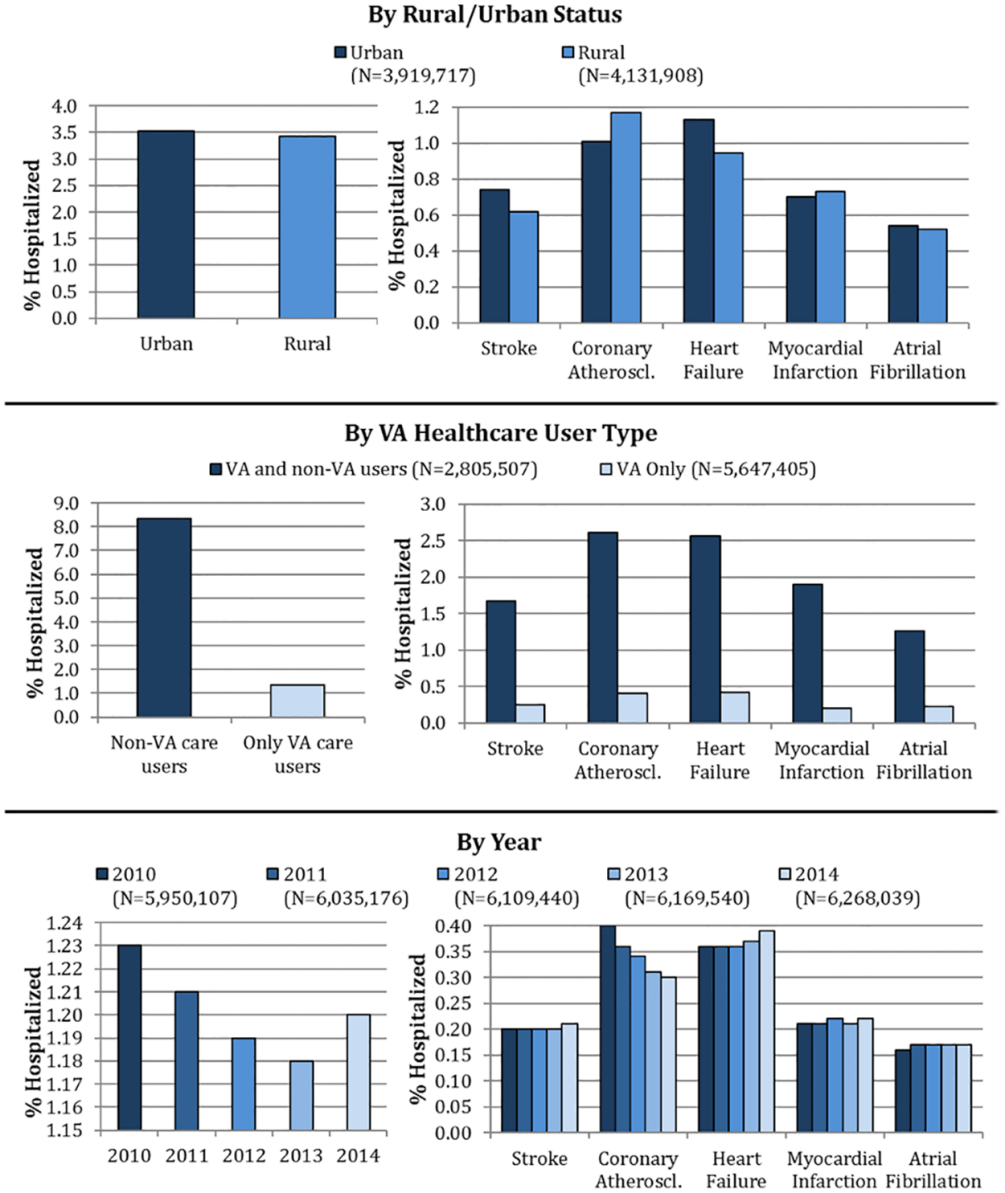
Variations in hospitalization rates by rurality, type of VA healthcare user and year. Coronary atheroscl.: Coronary atherosclerosis.

The age-adjusted proportion of veterans hospitalized for CVD was significantly higher for urban vs. rural veterans (3.5% vs. 3.4%, p<0.0001) [Fig 2, Table 3]. While atrial fibrillation and acute myocardial infarction seemed to affect rural and urban veterans similarly, urban veterans had higher age-adjusted rates of hospitalization for heart failure than rural veterans (1.13% vs. 0.94%). A multivariate regression model showed 19% greater odds of CV hospitalization among urban veterans compared to rural veterans (OR 1.19, p<0.0001) [Table 3]. Similarly greater odds were observed for urban veterans with multivariate models for each of the 5 CV conditions [Table 4].

### Temporal trend and VA healthcare user type

Temporally, the age-adjusted rate of CV hospitalization dropped from 1.23% in 2010 to 1.18% in 2013, but increased to 1.20% in 2014 [Fig 2]. While age-adjusted rates decreased consistently between 2010 and 2014 for coronary atherosclerosis, a slight increase was seen from 2013 to 2014 for stroke, heart failure, and myocardial infarction. On examination of the annual CV hospitalization rates from 2010 to 2014, we found a consistent decrease in age-adjusted rates between 2010 and 2013 followed by an increase in 2014 for a majority of the categories of sex, race, ethnicity, region and rural/urban status [Table 2]. The increase in CV hospitalization rate from 2013 to 2014 was particularly evident among blacks, American Indians, urban veterans and veterans living in the North Atlantic region [Table 2]. On adjusting for other covariates, we found overall lower odds of CV hospitalization in 2011–14 in comparison to 2010 [Table 3]. However, the odds of hospitalization for heart failure (OR 1.04, p<0.0001), myocardial infarction (OR 1.04, p<0.0001) and atrial fibrillation (OR 1.06, p<0.0001) were significantly higher in 2014 compared to 2010 [Table 4].

Adjusted for age, over the 5-year period, 8.3% of veterans who used non-VA care experienced one or more CV hospitalizations, in comparison to 1.3% of veterans who used only VA care [Fig 2]. This trend was consistent after adjustment for other demographics and veterans who used non-VA care were 4.98 times more likely than VA-only users to experience a CV hospitalization (95% CI 4.94, 5.02, p<0.0001) [Table 3]. Similar associations were also noted for each of the 5 individual conditions [Table 4].

### Region

**Fig 3:**
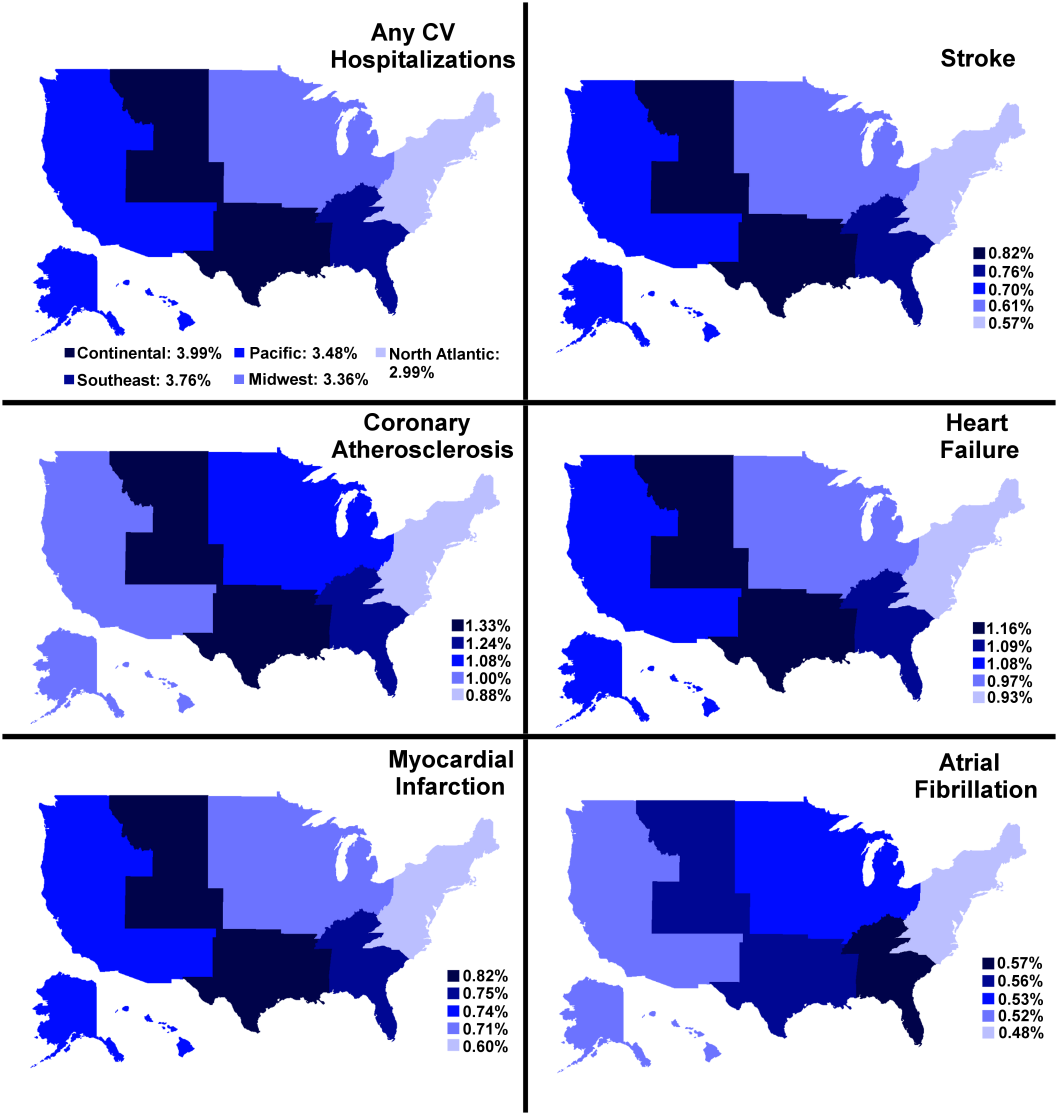
Variation in hospitalization rates by region. [Figure similar but not identical to the original image obtained from USGS National Map Viewer (open access) at http://viewer.nationalmap.gov/viewer/, and is therefore for illustrative purposes only].

Geographically, veterans living in the Continental region showed the highest age-adjusted CV hospitalization rate (3.99%) over the 5-year period, while veterans living in the North Atlantic region had the lowest rate (2.99%) [Fig 3]. Looking at regional differences by condition, the Continental region experienced the highest age-adjusted rates of hospitalization due to coronary atherosclerosis (1.33%), heart failure (1.16%), myocardial infarction (0.82%) and stroke (0.82%) [Fig 3]. The North Atlantic region experienced the lowest age-adjusted rates for all five conditions.

## Discussion

We sought to identify the top 5 causes of CV hospitalization in US veterans and to compare rates of CV hospitalization by age, sex, race, region, and year, using national electronic health records. Among 8,452,912 unique veterans who accessed VA healthcare during a 5-year period (Jan 2010–Dec 2014), the top 5 causes of CV hospitalization were: coronary atherosclerosis, heart failure, acute myocardial infarction, stroke and atrial fibrillation. Overall, 297,373 (3.5%) veterans were hospitalized for one or more of these cardiovascular conditions. However, there was significant variation in rates of CV hospitalization by gender, race, ethnicity, geographic region, urban vs. rural status, and year. In particular, older, male, Black, non-Hispanic, urban, and Continental region veterans experienced the highest rates of CV hospitalizations.

This is the first study using complete nationwide data (as opposed to a sample) to understand the patterns of cardiovascular hospitalization in the Veterans Health Administration on a large scale. The importance of our study pertains to the unique nature of both the veteran population and the Veterans Health Administration as a healthcare system. Since veterans have different health exposures and the Veterans Administration acts as a single payer system, one cannot presume that national trends such as those described previously[2] would apply to the veteran population. Over 8 million veterans receive healthcare through the Veterans Health Administration system every year[11], and cardiovascular disease is the leading cause of hospitalization[12]. Although many smaller studies have examined racial and gender differences[13–16], geographical variations[17, 18], temporal trends[19, 20] and utilization of healthcare services[21] pertaining to different aspects of the diagnosis, treatment, care and outcomes of patients with cardiovascular disease, population-wide descriptions of CVD epidemiology have only recently become possible due to the consolidation of national electronic health records in a centralized CDW.[3, 22] In an era where EHRs are becoming increasingly central to epidemiological research[23–25] and efforts are being made to standardize and share EHR data across health systems[26–30], the assembly of big data resources in a single repository provides a unique and unparalleled opportunity to study population-level trends in health and healthcare utilization. Moreover, the usefulness of EHRs in clinical research provides incentives to explore their use in clinical trials[31–33].

We found marked variance in rates of CV hospitalization by sex, race, and ethnicity. Odds of CV hospitalization were lower in women than men. A previous study showed that among people older than 65 years of age in 2010, women accounted for the majority of hospital stays for stroke[2, 34]. Although veterans with CV hospitalizations in our study were 68 years old on average, we found that male veterans demonstrated higher rates of stroke hospitalizations than females. Blacks had greater odds than whites of hospitalization for stroke or heart failure, but lower odds of hospitalization for coronary atherosclerosis or atrial fibrillation. Asians had the lowest rates of hospitalization for all 5 conditions. Sadly, the black vs. white difference in heart failure hospitalization was unchanged from a survey that was conducted more than 10 years ago[35]. Similarly, it was found that among Medicare beneficiaries, the rate of stroke hospitalization for blacks was 30% higher than for whites[2, 36]. We also found that whites had the highest rates of hospitalization for atrial fibrillation, similar to findings from a study using the National Hospital Discharge Survey data[2]. We also observed striking differences in rates of CV hospitalization by geographic region. Rates of CV hospitalization were higher in urban vs. rural veterans. As compared with veterans living in the Pacific region, rates of CV hospitalization were higher among those living in the Continental and Southeast regions. The most dramatic difference was in hospitalizations for coronary atherosclerosis: veterans in the Continental and Southeast were 32% and 23% respectively more likely than those in the Pacific region to be hospitalized. Unfortunately, these geographic patterns appear unchanged from those observed over 20 years ago among veterans admitted with cardiovascular diagnoses.[13] Similar studies of Medicare beneficiaries have found that rates of hospitalization for acute MI and heart failure were higher in the Southeast than in the West[35, 37], suggesting that regional differences in CV health are stable across patient populations in the US. Future studies are needed to determine whether these differences are due to variation in clinical practices or to demographic factors themselves.

Finally, we observed a decrease in age-adjusted rates of CV hospitalization between 2010 and 2013 followed by a slight increase in 2014. Broken down by condition, the increase from 2013 to 2014 appears to be driven by stroke, heart failure and myocardial infarction. The increase in overall CV hospitalization rate from 2013 to 2014 was also evident among blacks, American Indians, urban veterans and veterans living in the North Atlantic region. Previous studies have shown that the absolute number of hospital discharges for cardiovascular disease in the US decreased from 2000–2010.[2] However, the number of inpatient discharges for stroke increased during the same time period while those for heart failure remained unchanged[2]. Examination of hospitalization rates among patients aged 65 and above for coronary heart disease from the National Hospital Discharge Surveys showed a decrease between 1980 and 2006.[38] While our findings somewhat match national trends, the increase in hospitalization rates from 2013 to 2014 is concerning. Future research is needed to determine whether this is an ongoing trend and if so, what patient subpopulations are most affected and the causes for such increase.

Several limitations must be kept in mind when interpreting our results. First, it is possible that different coding practices across VA medical centers might contribute to some of the geographical variations that we observed. Second, electronic health records have many inaccuracies[3, 39–41]. Since we did not use chart review to document CV hospitalization, misclassification of the reasons for CV hospitalization is a possibility. Third, the rates only reflect hospitalizations within the VA healthcare system, and not all veterans are enrolled in the VA healthcare system. Therefore, the results may have limited generalizability. Finally, we were unable to determine reasons for the variations in hospitalization rates by gender, race, ethnicity, region, and year. It is possible that these observations reflect differences in comorbidity or socioeconomic status across the population or regional clinical practices.

In summary, the adoption of electronic records has substantially improved our ability to evaluate population-level healthcare patterns. Variations in hospitalization rates by demographic and geographic factors could signal differential access to care, disparities in quality of care, differential distribution of risk factors or variations in genetic susceptibility to disease. Future studies should aim to determine what exposures and risk factors account for the high rates of cardiovascular disease in these subpopulations. The use of national data to determine gender, racial and regional variations in healthcare will inform future healthcare policy and allocation of resources.
