# Correction: Leading causes of cardiovascular hospitalization in 8.45 million US veterans

**DOI:** 10.1371/journal.pone.0200327

**Published:** 2018-07-03

**Authors:** Nirupama Krishnamurthi, Joseph Francis, Stephan D. Fihn, Craig S. Meyer, Mary A. Whooley

There is an error in Table 1. The values in the “All” column are incorrect. The correct Table 1 can be found below.

**Table 1:** Characteristics of veterans hospitalized vs. not hospitalized for one or more of the top 5 cardiovascular conditions between 2010 and 2014 *†.

Patient Characteristics		All		Hospitalized		Not Hospitalized	
		N = 8,452,912		N = 297,373		N = 8,155,539	
Age, years (mean ± SD)		60.01 ± 17.63		68.24 ± 11.18		59.71 ± 17.75	
Sex	Female, number (%)	579,623	6.9	7,045	2.4	572,578	7.0
	Male	7,873,289	93.1	290,328	97.6	7,582,961	93.0
Race	White	5,848,351	69.2	223,778	75.3	5,624,573	69.0
	Black or African American	1,203,447	14.2	47,910	16.1	1,155,537	14.2
	Native Hawaiian or Other Pacific Islander	61,293	0.7	2,108	0.7	59,185	0.7
	American Indian or Alaska Native	55,191	0.7	1,849	0.6	53,342	0.7
	Asian	75,325	0.9	1,330	0.5	73,995	0.9
Ethnicity	Not Hispanic	7,131,212	84.4	270,873	91.1	6,860,339	84.1
	Hispanic	452,100	5.3	14,751	5.0	437,349	5.4
Region	Southeast	1,646,007	19.5	61,067	20.5	1,584,940	19.4
	Midwest	1,786,834	21.1	62,495	21.0	1,724,339	21.1
	North Atlantic	1,850,516	21.9	57,662	19.4	1,792,854	22.0
	Continental	1,340,866	15.9	50,453	17.0	1,290,413	15.8
	Pacific	1,431,580	16.9	47,962	16.1	1,383,618	17.0
Rural/Urban Status	Urban	3,919,717	46.4	136,450	45.9	3,783,267	46.4
	Rural	4,131,908	48.9	143,114	48.1	3,988,794	48.9
VA Healthcare User Type	VA Only	5,647,405	66.8	79,180	26.6	5,568,225	68.3
	VA and Non-VA Users	2,805,507	33.2	218,193	73.4	2,587,314	31.7
Diagnosis	Coronary arteriosclerosis	93,380	1.1	93,380	31.4		N/A
	Heart failure	88,769	1.1	88,769	29.9		
	Acute myocardial infarction	61,501	0.7	61,501	20.7		
	Stroke	58,386	0.7	58,386	19.6		
	Atrial fibrillation	45,115	0.5	45,115	15.2		

* All p values (hospitalized vs. not hospitalized) <0.0001 except rural/urban status (p = 0.3967).

† Unknown race: 20,398 (6.9%) hospitalized; 1,188,907 (14.6%) not hospitalized

Unknown ethnicity: 11,749 (4.0%) hospitalized; 857,851 (10.5%) not hospitalized

Unknown region: 17,734 (6.0%) hospitalized, 379,375 (4.7%) not hospitalized

Unknown rural/urban status: 17,809 (6.0%) hospitalized; 383,478 (4.7%) not hospitalized
